# Dogs' Social Referencing towards Owners and Strangers

**DOI:** 10.1371/journal.pone.0047653

**Published:** 2012-10-11

**Authors:** Isabella Merola, Emanuela Prato-Previde, Sarah Marshall-Pescini

**Affiliations:** Section of Psychology, Department of Biomedical Sciences and Technologies, University of Milan, Milan, Italy; Tulane University Medical School, United States of America

## Abstract

Social referencing is a process whereby an individual uses the emotional information provided by an informant about a novel object/stimulus to guide his/her own future behaviour towards it. In this study adult dogs were tested in a social referencing paradigm involving a potentially scary object with either their owner or a stranger acting as the informant and delivering either a positive or negative emotional message. The aim was to evaluate the influence of the informant's identity on the dogs' referential looking behaviour and behavioural regulation when the message was delivered using only vocal and facial emotional expressions. Results show that most dogs looked referentially at the informant, regardless of his/her identity. Furthermore, when the owner acted as the informant dogs that received a positive emotional message changed their behaviour, looking at him/her more often and spending more time approaching the object and close to it; conversely, dogs that were given a negative message took longer to approach the object and to interact with it. Fewer differences in the dog's behaviour emerged when the informant was the stranger, suggesting that the dog-informant relationship may influence the dog's behavioural regulation. Results are discussed in relation to studies on human-dog communication, attachment, mood modification and joint attention.

## Introduction

Social referencing is a process whereby individuals use another's emotional cue towards a novel object/event to guide their own future behaviour towards it [1]. From a functional perspective, the importance of social referencing is that, like all social learning processes, it allows an individual to avoid making costly errors associated with trial-and-error learning [2]. Social referencing includes two distinct components: the subject's referential looking at the informant (i.e. looks immediately preceded and/or followed by a look to the novel object), and the subject's behavioural regulation based on the emotional information received from the informant [2]. Many studies have shown social referencing in toddlers and infants [3]–[6]. Overall results show that infants look at the informant (generally their care-giver) and change their behaviour according to the emotional messages received: when receiving a positive message they reach closer to the object and interact with it faster than when receiving a negative one [7]–[9]; conversely when negative emotional information is conveyed they play less with the toy, look longer/more frequently at the care-giver, and move slower towards the care-giver [10], .

Studies have also looked at social referencing in infants when the emotional message towards an ambiguous object was conveyed either by a stranger or a familiar person [8], [12], [13]–[15]. According to a number of authors, the fact that infants under circumstances of ambiguity look at a stranger as much as at the care-giver (acting as the informant) shows that referential looking is not a mere form of comfort seeking, but rather the search for information about the specific situation [14], [16]. In fact a number of studies have shown that referential looking occurs equally with a stranger or the mother acting as the informant [8], [9], [12], [13], [17]. However, behavioural regulation in accordance with the stranger's emotional message occurs only if the mother is also present in the room (presumably because she serves as a ‘secure base’ [9]); in this case infants approach the mother more when fear signals are being delivered, whereas they approach the object more when receiving a positive message from the stranger [12]. But if infants are alone with the stranger they do not regulate their behaviour, suggesting that this process may vary according to the relationship with the informant and the presence of a bonded figure [13].

There is mixed evidence of social referencing in other species. A number of studies [18], [19] found no evidence of referential looking in captive mother-infant pairs of chimpanzees and infant Barbary macaques. However, other studies [2], [20] found evidence of some aspects of social referencing in chimpanzees. In one study [2], human-reared chimpanzees showed referential looking towards their human caregiver and looked longer at the objects when a happy message was delivered, whereas they withdrew from the object more frequently when receiving a fearful message. In the other study [20], infant chimpanzees looked towards and returned to their mother when the object was first presented: however, it was not possible to establish whether behavioural regulation based only on the voice and facial expression of the mother occurred since her movements were not restricted. Finally, capuchin monkeys have been shown to appropriately associate the emotional valence of a conspecific's expression towards an object [21]. Having observed a conspecific open two identical boxes, which either elicited a positive or a negative reaction, subjects approached the ‘positive box’.

In a previous study, we found good evidence that domestic dogs look referentially towards the owner when confronted with an ambiguous object, but there was only slight evidence of behavioural regulation [22]. This paucity of results in terms of behavioural regulation is somewhat surprising given that dogs have been shown to: (i), discriminate between smiling and neutral human faces [23] and potentially also more diverse facial expressions [24]; (ii), be positively influenced by a human demonstrator talking, both in a social learning task [25] and in a classic two-object choice pointing task [26]; and (iii), be sensitive to the tone of voice (gentle vs. harsh) used by a human in an obedience task [27], a pointing task [28], and when evaluating a third party interaction in a begging paradigm [29]. Taken together these findings suggest that dogs have at least some basic sensitivity towards humans' emotional messages, even when these are conveyed only through facial and vocal means.

Thus, the limited behavioural regulation that emerged in our previous study may have been caused by small, but potentially important differences between our procedure and that used to test infants. In infant studies mothers immediately deliver the emotional message after their child looks at them; furthermore, towards the end of the test the ‘noisy/movable scary toy’ is normally switched off whilst the mother continues delivering her message [3], [7], [30], making it less intimidating for the child to eventually approach. In our previous study, owners were asked to stay silent for the first 15 seconds of the test, regardless of the dogs' looking behaviour. This allowed us to assess whether dogs would look back to the owner not only to obtain food or a desired toy (as has been shown in numerous studies [31]–[35]), but also when facing a new and potentially scary object. However, this procedure implied that the first time dogs looked at the owner they received no overt response, which may have conveyed an unclear message about the value of the object. Furthermore, owners did not alternate their gaze between the dogs and the object, omitting a potentially important cue displaying the communicative intent of the informant [26]. Finally, differently from infant studies, our ‘strange object’ was switched off only at the end of the test. Hence, we were unable to evaluate whether, when the stimulus is made less scary and the informant continues delivering the emotional message, the dog's behaviour changes in accordance with the emotion expressed.

The first aim of the current study was to assess whether when facing an ambiguous stimulus dogs, like infants [8], [9], [12], [13], [17], will use referential looking towards the informant regardless of their level of familiarity (stranger vs. owner). Based on infant studies, this would allow us to show that the dogs' looking behaviour cannot be explained in terms of comfort seeking from the attachment figure, but represents a search for information from the person actively involved in the situation. The second aim was to test dogs with a social referencing procedure closely mirroring that used with infants, to evaluate whether the poverty of the behavioural regulation response observed in the previous study with the owner as the informant may have been due to methodological differences.

Finally, we aimed at assessing whether behavioural regulation would vary according to the dog's relationship with the informant (stranger vs. owner). A number of studies suggest that dogs form a strong attachment bond with their owners, similar to the human mother-infant relationship [36], [37], and that, like children, they use their owner as a ‘secure base’ [38]. Furthermore, two studies indicate that dogs' comprehension and use of communicative cues is influenced by the identity of the informant/recipient. In one study, dogs were more likely to inform their owner than a stranger about the location of a hidden object which was of interest only to the person [39]; in the other, dogs that received a pointing cue to an empty container from their owner compared to a stranger, took longer to extinguish their response when the owner was performing the cuing task [40]. There is also some evidence that the quality of the dog-owner bond may affect the dogs' problem solving abilities [41], [42], and that in some situations dogs show clear preferential visual attention towards their owner [43]. Taken together these results suggest that, at least in some situations, dogs show differential behaviours depending on the identity of the person they observe or interact with.

In the current study, to assess the influence of the informant's identity on dogs' referential looking, either the owner or the stranger acted as the informant (whilst the non-acting person sat quietly in the testing room, reading a magazine). To evaluate the presence of behavioural regulation, dogs' behaviour was measured when the informant delivered the message (positive or negative) about the ambiguous stimulus that, following the infant procedure, was subsequently switched off. Hence, dog-owner dyads were randomly assigned to one of four groups: owner-positive, owner-negative, stranger-positive, stranger-negative. Between-groups comparison allowed us to assess the presence of referential looking and behavioural regulation and whether they differed according to the identity of the informant.

Given dogs' use of referential looking to the owner in a social referencing paradigm [2] and the use of gaze alternation as a communicative tool also towards strangers in a variety of requesting situations [31]–[34], [39], [44], we hypothesized that dogs would use referential looking also towards a stranger when confronted with a novel, ambiguous object. Furthermore, considering the evidence of some behavioural regulation in our previous social referencing study [22] and the procedural modifications of the current study, we hypothesized a differential pattern of behaviour for dogs in the positive vs. negative message groups. More specifically we predicted that, similarly to infants, dogs in the negative message groups (owner-negative, stranger-negative) would look at the informant more often, stay further away from the object, and generally move less than those in the positive message groups (owner-positive, stranger-positive), whereas dogs in the latter groups would move closer to the object and interact with it more (especially when it was turned off). Finally, considering previous studies on the dog-owner relationship, we expected a differential pattern of behaviours in dogs tested with the stranger as the informant, compared to dogs tested with the owner as the informant. In line with the infant literature, we predicted that both with the owner and stranger acting as informant dogs would approach the object more in the positive than the negative group, but they would stay closer to the owner in the negative message groups.

## Methods

### Ethics Statement

No special permission for use of animals (dogs) in such socio-cognitive studies is required in Italy. The relevant ethical committee is the Ethical Committee of the Università degli Studi di Milano. All the owners who visit our lab with their dogs sign a consent form and each time they visit for a new behavioural study they are carefully briefed to obtain consent for participation.

### Subjects

Ninety dogs (37 males, 53 females; mean 4.7 years SD 3.29 range: 1–13; 61 pure breed, 29 mixed breed- see Text S1) and their owners participated in the study. Dog-owner dyads were semi-randomly assigned to one of four groups, balancing for sex and age. Thus, 44 dogs participated in the study with their owners as the informant: of these 26 were tested with the owner conveying a positive emotional message (owner-positive group) and 18 with the owner giving a negative emotional message (owner-negative group) about the object. Forty-six dogs were tested with the same female stranger (IM) acting as the informant: of these 21 witnessed the stranger giving a positive message (stranger-positive group) and 25 a negative message (stranger-negative group). All dogs were pets and lived at home with their owners.

#### Stimulus Selection

**Figure 1:**
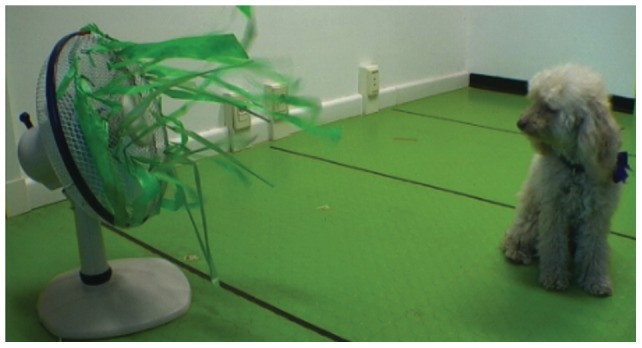
Experimental object. A fan with plastic green ribbons attached to it (and a curious subject).

The experimental stimulus was the same for all dogs in all groups: a 50 cm tall and 34 cm wide electric fan, with plastic green ribbons attached to it (Figure 1). This stimulus was selected in our previous study [22] because it elicited a mild fear reaction, similarly to stimuli used in infant studies [3], [7]. This object evokes in most dogs a cautious reaction, i.e. neither very positive (approaching directly and touching) nor very negative (running in the opposite direction or strong stress such as trembling, or hiding).

### Procedure

The dogs were individually tested in an unfamiliar (2.5×3.5 m) room of the laboratory *Canis Sapiens* of the University of Milan. On arrival dogs were given 5 minutes to freely explore the empty testing room, while the experimenter explained the procedure to the owner. During this time the experimenter ignored the dog completely.

**Figure 2:**
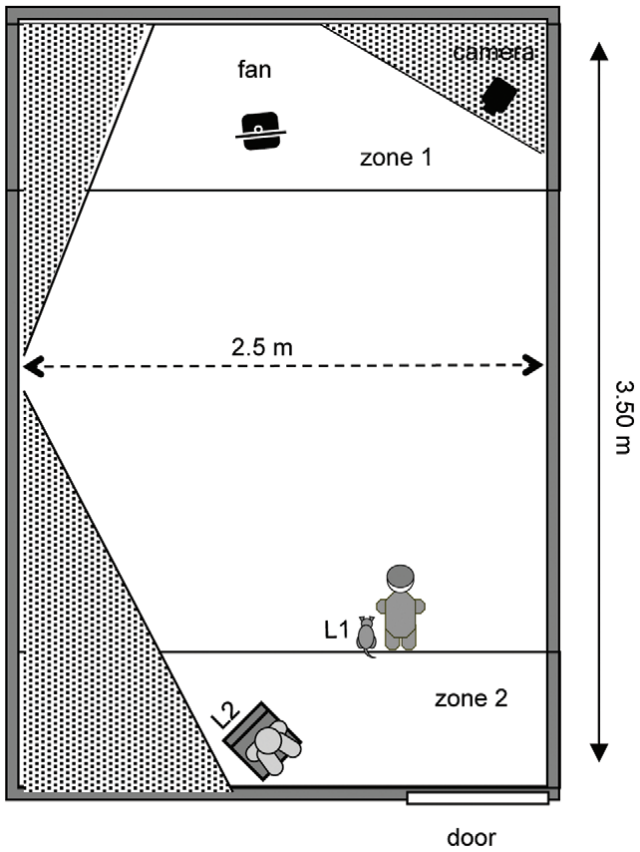
Experimental set up. The experimental room showing the Fan-zone (Zone 1: 230×85cm) and the Door-zone (Zone 2: 230×85cm). The dog is represented next to the informant (the standing person) in the location where it was first released (L1). Both the informant and the seated person remained in the same position throughout the test.

The test lasted 50 seconds and was divided into two phases lasting 25 seconds each. During the entire test the fan remained placed at the far end of the room (see Figure 2).

Dogs were tested either with the owner or with the stranger conveying either a positive or negative message towards the fan. Owner and stranger were always both present in the room (as in infant studies e.g. [12]), however the person who was not acting sat quietly in a chair facing away from the fan/dog and reading a magazine for the entire duration of the test.

Each dog was allocated to one group only and thus exposed either to the positive or negative message, with either the stranger or owner delivering it.

The test phases were identical for all groups: Phase 1 (Ph 1): the informant entered the room holding the dog by its collar and stopped at location 1. At the same time the other person (owner or stranger depending on group allocation) sat on a chair in the room reading a book with their back to the fan (at location 2), without moving until the end of the test. As soon as the informant closed the door, the fan was activated by remote control. The informant and dog stopped at location 1, facing the fan, where the dog was released and allowed to move freely around the room. The informant remained silent looking at the fan until the dog looked back at her/him the first time. From this moment the informant started to respond alternating their gaze between the dog and the fan every time the dog looked at her/him, and, depending on group allocation, using either a happy (positive message) or fearful (negative message) voice and facial expression. Phase 2 (Ph 2): the experimenter turned the fan off using the remote control. The informant whilst remaining in the same position (location 1) continued to respond to the dog every time it looked at her/him, using either a happy (positive message) or a fearful (negative message) voice and facial expression.

In both the positive and negative group, in phases 1 and 2 the owners and stranger were instructed to deliver their message only when the dogs were looking at them. They were also asked to alternate their gaze between the object and the dog whilst delivering the message and to communicate using typical phrases such as “that's lovely”, “so beautiful” or “that's ugly”, “that's scary”, accompanied by either a smiley happy face or a scared worried expression. They were explicitly told not to use the dog's name and potential commands such as “look, go, come, touch, away”. They were instructed to convey, through facial and vocal expression, the feeling either that the dog could safely and happily approach the object or that the object was dangerous and fearsome for the dog. After the test ended the experimenter went out of the room to get some pieces of food, and together with the owner sat next to the fan, giving the dog treats when it came in proximity of the fan. All dogs received this treatment so that they would not become sensitive to fans.

### Data collection and analysis

The test was recorded by a video camera (Panasonic NV-GS330), and analysed using Solomon Coder (beta 081122, Copyright 2006–2008 by Andràs Péter). All the statistical analyses were carried out with IBM SPSS Statistics 19.

Following [2], referential looking was defined as a gaze towards the informant that was preceded/followed -within 2 seconds- by a look to the fan and gaze alternation as a consecutive sequence of three looking behaviours (fan-informant-fan or informant-fan-informant). Referential looking was analyzed only in Ph 1, whereas the latency to interact and reach the fan–zone only in Ph 2. All the other behaviours were analyzed in both phases. Two non-mutually exclusive categories of behaviour were recorded: Action and Gaze. Furthermore, the location of dogs in two areas of the room, the Fan-zone and the Door-zone, was recorded (Table 1). The Fan-zone (2.30×85 cm) was the area closest to the fan and the Door-zone (2.30×85 cm) the area furthest from the fan (see Figure 2).

**Table 1:** Behavioural categories.

**ACTION**	
Interact fan	the dog is in physical contact with the fan
Interact informant	the dog is in physical contact with the informant
Interact seated person	the dog is in physical contact with the seated person
Static	The dog is in any position which does not involve movement i.e. standing, sitting or lying
Locomotion	the dog is in motion e.g. exploration of the room, approaching a person or simply walking around
Approach fan	the dog's face is oriented towards the fan and there is a reduction in the distance between itself and the fan
**GAZE**	
Gaze seated person	the dog's head is oriented towards the person that was inactive during the test
Gaze informant	the dog's head is oriented towards the person that was delivering the message (positive or negative)
**ZONE**	
Door zone	An area of 230×85cm closest to the door and farthest from the fan
Fan zone	An area of 230×85cm closest to the fan and farthest from the door

Three non-mutually exclusive categories were used: action, gaze and areas of the room used by the dogs; within each category mutually exclusive behaviours and their descriptions are outlined.

The dogs' behaviour was coded from video by the first author (IM). A second independent blind coder (SMP) analysed 25% of the data and Spearman correlations were calculated for the main behavioural categories (Gaze Own: r = 0.79, p = 0.000; Gaze Exp: r = 0.83, p = 0.000; Approaching Fan: r = 0.95, p = 0.000; Door Zone r = 0.93, p = 0.000).

To evaluate whether informant identity, message valence and test phase affected the dogs' behaviours a Generalized Estimating Equation (GEE) with Bonferroni corrected posthoc tests was used with the following predictor variables: informant (owner vs. stranger), message (positive vs. negative) and test phase (Ph 1 and Ph 2). The frequency of gazing at the owner and the stranger and the duration of all the actions, and zone use (see Table 1) were used as dependent variables.

Furthermore, latencies to reach the Fan-zone and Interact with the fan in phase 2 of the test were analyzed using a Generalized Linear Model (GLM) with Bonferroni corrected posthoc tests with the informant (owner vs. stranger) and message (positive vs. negative) as predictor variables. The same model was used to compare the duration of the messages delivered by the informant in the 4 groups, and to compare the frequencies of gaze alternation between fan and the owner when s/he was the informant vs. the seated person.

Chi-square tests were used to compare the number of dogs that showed referential looking and gaze alternation towards the informant in the owner vs. stranger group and the number of dogs that interacted with the fan in the positive vs. negative message group. Finally, a Wilcoxon test was used to compare the frequency of gazing at the informant vs. the seated person.

## Results

Of the ninety dogs tested, eight dogs (2 males and 6 females) were excluded from all analyses, because of procedural errors committed by the owners during testing.

Of the remaining eighty-two dogs, twenty-five (14 males and11 females) approached and touched the fan during the first 25 seconds of the test (Ph 1), exhibiting a confident and positive attitude towards the stimulus. These dogs were excluded from further analyses of social referencing, since a pre-condition for this test is that dogs show an ambiguous (or mildly fearful) behaviour towards the stimulus object, and because the more experience a subject has had with a particular object the less receptive he will be to social referencing regarding that object [11], [45], [46].

Of the remaining fifty–seven dogs, 3 never looked back at the informant, and hence never received a message. These dogs were included in the analyses for referential looking and gaze alternation but, in line with the approach taken by [17] and [9] in their infant studies, they were excluded from the analyses of behavioural regulation.

### Referential looking and Gaze alternation

To assess whether dogs carried out referential looking and gaze alternation towards the informant when confronted with an ambiguous stimulus we analysed dogs' gazing behaviour in Ph 1. Twenty-two of 29 (76%) dogs in the owner group (positive and negative) and 17 of 28 (60%) dogs in the stranger group (positive and negative) showed referential looking towards the informant. This difference was shown not to be significant (χ = 1.5; p = 0.22).

Gaze alternation, defined as a 3-way interaction (i.e. person-fan-person or fan-person-fan), was coded both between the object and the informant and between the object and the seated person. Considering positive and negative message groups together, gaze alternation between fan and informant was shown by 18 dogs in the owner-informant group (62%) and 15 dogs in the stranger-informant group (52%). This difference was shown not to be significant (χ = 0.4; p = 0.5). Conversely, considering positive and negative message groups together, gaze alternation between the fan and the seated person was exhibited by 12 dogs in the owner-seated group (37%) and 2 dog in the stranger-seated group (3%). This difference was shown to be significant (χ = 9.94; p = 0.002).

To assess whether dogs took into consideration the attentiveness of the person, we compared the frequencies of gaze alternation between fan and the owner when s/he was the informant vs. the seated person. Dogs' gaze alternation was significantly higher when the owner was the informant than when s/he was seated and inattentive (mean informant  = 1.7, seated  = 0.5, Wald  = 15, p<0.001). The same analysis was carried out when the stranger was either the informant or the seated person and similar results emerged (mean informant  = 0.9, seated  = 0.24, Wald  = 8.7, p = 0.003).

To evaluate whether the dogs' looking behaviour was directed specifically to the informant, gaze frequency to the informant and the seated person were compared for the stranger/informant group and the owner/informant group separately. In the stranger-informant group dogs looked at the seated owner and stranger equally (Wilcoxon z = 0.9, p = 0.4), whereas in the owner-informant group dogs looked significantly more at the owner than the seated stranger (Wilcoxon z = 3.9, p<0.001).

### Behavioural regulation

Having established that dogs use referential looking also towards a stranger when confronted with an ambiguous object, we assessed whether the dogs' reaction would be affected by the valence of the emotional expression delivered and by the informant's identity.

Of the 54 dogs that showed an ambiguous approach towards the fan in Ph1, twenty–seven (10 males and 17 females) were in the owner group and twenty–seven (10 males and 17 females) in the stranger group. In the owner group fourteen dogs (5 males and 9 females) were tested with the positive message and thirteen (5 males and 8 females) with the negative message; in the stranger group twelve dogs (5 males and 7 females) were tested with the positive message and fifteen (5 males and 10 females) with the negative message.

**Figure 3:**
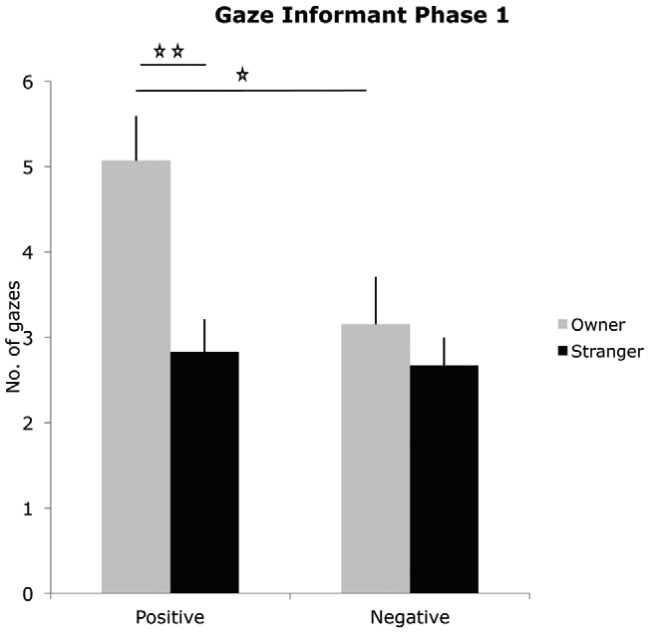
Gaze informant. Mean frequency of gazes directed towards the informant during Phase 1 for dogs in the owner-positive, owner-negative, stranger-positive and stranger-negative groups. The bar represents the standard error (SE).

Significant differences emerged in Gazing towards the informant (GEE informant × message × test phase, Wald = 43.4, p<0.001, see Figure 3) and in Gazing towards the seated person (GEE informant × message × test phase Wald = 29.32 p<0.001). In all groups dogs looked at the informant more often in Ph 1 than Ph 2 (stranger-positive: phase 1 vs. 2, p<0.001; stranger-negative: phase 1 vs. 2, p = 0.018; owner-positive: phase 1 vs. 2 p =  0.003; owner-negative: phase 1 vs. 2 p = 0.01). In the positive message group dogs gazed at the informant more often if s/he was the owner rather than the stranger; this occurred in both phases (phase 1: mean owner  = 5.07 vs. stranger  = 2.83, p<0.001; phase 2: mean owner  = 2.57 vs. stranger  = 1, p = 0.01). When the informant was the owner, dogs in the positive message group looked at him/her more often than dogs in the negative message group (mean owner-positive  = 5.07 vs. mean owner-negative  = 3.15, p = 0.01) but only in Ph 1. No such difference emerged in the stranger group. In the negative message group dogs looked at the seated person more often if s/he was the owner rather than the stranger (mean owner  = 2.17 vs. mean stranger 1, p<0.001) but only in Ph 1.

**Figure 4:**
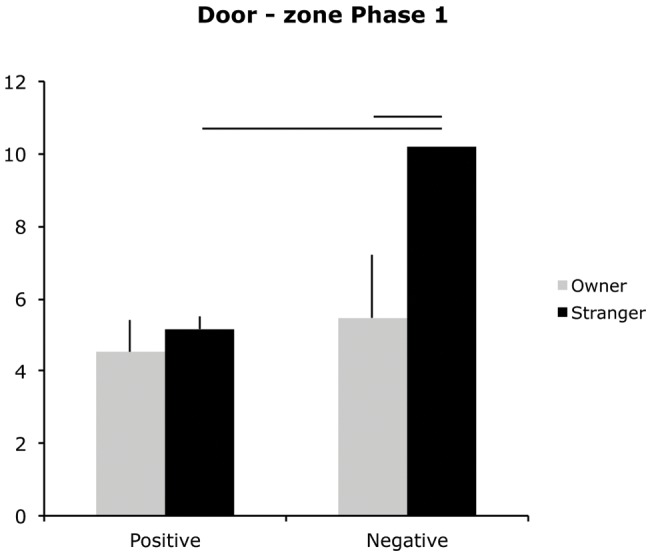
Door – zone. Mean duration (in seconds) of time spent closest to the door (hence farthest from the fan) in Phase 1 for dogs in the owner-positive, stranger-positive and owner-negative, stranger-negative groups. The bar represents the standard error (SE); * p<0.05, **p<0.001.

**Figure 5:**
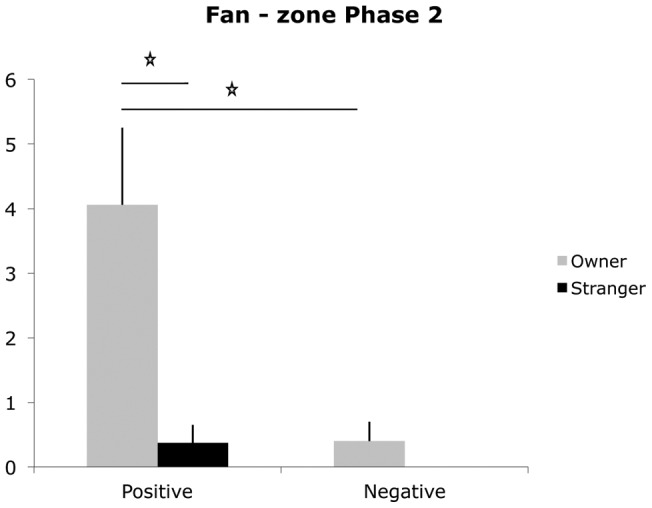
Fan – zone. Mean duration (in seconds) of time spent closest to the fan in Phase 2 for dogs in the owner-positive, stranger-positive, owner-negative and stranger-negative groups. The bar represents the standard error (SE); * p<0.05, **p<0.001.

Significant differences emerged in the time spent in the Door-zone (farthest from the fan) (GEE informant × message × test Wald = 16.52, p = 0.02) and in the Fan-zone (closest to the fan) (GEE informant × message × test Wald = 18.77, p = 0.005) (Figure 4 and 5). When the informant was the stranger, dogs in the negative message group spent more time in the Door-zone compared to dogs in the positive message group in both phases (Ph 1: mean negative group  = 10.18 vs. positive group  = 5.16, p = 0.02; Ph 2: mean negative group  = 14.44 vs. mean positive group  = 3.75, p = 0.002). In Ph 1, dogs that received a negative message, spent longer in the Door-zone when the informant was the stranger than when s/he was the owner (mean stranger-negative group  = 10.18 vs. owner-negative group  = 5.47, p = 0.043). During Ph 2 dogs in the positive message group, spent more time in the Fan-zone if the informant was the owner rather than the stranger (mean owner-positive  = 4.06 vs. stranger-positive  = 0.37, p = 0.003). Furthermore, in the group of dogs tested with the owner as the informant, dogs receiving a positive message spent more time in the Fan-zone than dogs receiving the negative message (mean owner-positive  = 4.06 vs. owner-negative  = 0.4, p = 0.003).

Significant differences emerged in Approaching the fan (GEE informant × message × test Wald = 83.97, p = 0.001). During Ph 2 dogs in the positive message group, spent more time approaching the fan if the informant was the owner than if it was the stranger (mean owner-positive  = 2.75 vs. stranger-positive  = 1.1 p = 0.045). Furthermore, in the group of dogs tested with the owner as the informant, dogs receiving a positive message spent longer approaching the fan than dogs receiving a negative message (mean owner-positive  = 2.75 vs. owner-negative  = 0.64, p = 0.002).

There were also differences in Static behaviour (GEE informant × message × test Wald = 32.72, p = 0.001). In Ph 2, dogs tested with the stranger as the informant spent more time being static if the message they received was negative than if it was positive (mean stranger-positive  = 10.81 vs. stranger-negative  = 18.25, p = 0.01). An overall difference emerged in the dogs' frequency to interact with the seated person (GEE informant × message × test Wald = 14.35, p = 0.045) and with the informant (GEE informant × message × test Wald = 14.30, p = 0.03), but subsequent post-hoc tests were unable to detect where these differences occurred.

Furthermore, in Ph 2 significant differences emerged in the latency to reach the Fan-zone in relation to informant identity (Wald 9.14, p = 0.002) and message valence (Wald 13.89, p<0.001). When the informant was the owner, dogs in the positive message group reached the Fan-zone faster than with the stranger as the informant (mean owner-positive  = 28.84 vs. stranger-positive  = 50.97, p = 0.005). Furthermore, when the informant was the owner, dogs in the negative group took longer to enter the Fan-zone than dogs in the positive group (mean owner-positive  = 28.82 vs. owner-negative  = 54.21, p = 0.001). Moreover, in this phase significant differences emerged in the latency to interact with the fan in relation to informant identity (Wald 10.98 p = 0.001) and message valence (Wald 4.78 p = 0.029). In the positive message group dogs tested with the owner as the informant touched the fan sooner compared with dogs tested with the stranger as the informant (mean owner-positive  = 35.1 vs. stranger-positive  = 60, p = 0.001). Furthermore, when the informant was the owner, dogs in the negative group took longer to interact with the fan than dogs in the positive group (mean owner-positive  = 35.1 vs. owner-negative  = 55.44, p = 0.002).

In the positive message groups a greater number of dogs interacted with the fan when the informant was the owner rather than the stranger (Fisher exact: owner group  = 8 vs. stranger group  = 0, χ^2^ = 9.39, p = 0.002); no such difference was found between negative message groups (Fisher exact owner group  = 3 vs. stranger group 1, χ^2^ = 1.08, p = 0.24) where very few dogs touched the fan.

Finally, to assess whether the different patterns of behaviour in the positive and negative message groups may have been caused by the different amount of time spent delivering the messages, we compared mean duration of messages in the four groups: no significant differences emerged (Wald = 1.85, p = 0.6).

## Discussion

Social referencing is a process that could be useful in a variety of everyday life situations, such as meeting a new person, facing a new and ambiguous situation or a strange object. Given the dependent nature of dogs' relationship to humans [36], [37] adult dogs, like young children, may benefit from the ability to assess people's reaction to novel situations/stimuli and act accordingly. The aim of the current study was to assess the potential presence of social referencing in dog-human interactions. Given our previous study on this topic demonstrating the presence of referential looking towards the owner [22], we investigated the potential presence of this behaviour also towards a stranger; furthermore, using the same procedure adopted in infants' studies, we aimed at assessing the presence of behavioural regulation based only on the owners'/strangers' vocal and facial emotional reactions to the object, and evaluated potential differences in the dogs' reaction to the message depending on informant identity.

A number of studies have reported functionally referential communication in dogs, indicating that dogs use gaze and gaze alternation as a communicative tool in a variety of situations in order to request for out of reach toys or food [31]–[35], [39], [44]. Preliminary evidence also suggests that dogs, besides using gaze for requesting purposes, look at their owners to monitor their reaction to a strange object [22]. Current results confirm those of our previous study, with 76% of dogs looking back to the owner when confronted with a strange object, and extends them by showing that this behaviour occurs equally frequently when a stranger acts as the informant (60% of dogs looking back to the stranger). The pattern of gaze alternation between informant and ambiguous object is also unaffected by informant identity (62% owner vs. 52% stranger). These findings are similar to those emerging from the infant social referencing literature and showing that, in a similar situation, infants look referentially towards their mother (88%) but also towards a stranger (83%) or a familiar care-taker (86%) [8], [12], [17]. According to a number of authors [14], [16] looking at a stranger as much as at a familiar care-giver (acting as the informant) indicates that looking behaviour cannot be considered just a form of comfort seeking due to the activation of the attachment system, but rather it should be interpreted as a search for information about the specific context.

In a subsequent study with infants, however, a different set up was used to assess whether infants would preferentially look at a stranger actively informing them about the situation or at the inattentive mother, when both were present in the room [15]. Also in this scenario infants preferred looking at the active stranger, further excluding the possibility that looking was a comfort-seeking behaviour. In contrast, results from our study show that when the informant is a stranger and the owner is inattentive, dogs look at both to the same extent. Hence, differently from infants, dogs seem to look at the stranger-informant but also seek out the owner by looking towards him/her. Whether this behaviour is aimed at obtaining information also from the owner, or is a form of comfort seeking, remains an open question.

A further objective of this study was to examine the influence of the informant's vocal and facial expression on the dogs' behaviour towards the ambiguous object (the behavioural regulation aspect of social referencing). Results showed that dogs were affected by the positive vs. negative message received but in different ways according to the informant's identity. When the owner acted as the informant dogs in the positive group looked at him/her more often than dogs in the negative group, and also spent more time approaching the fan and in the Fan–zone. Conversely, dogs in the negative group took longer to reach the Fan-zone and interact with the fan. These findings are in many ways similar to those found in infants. Indeed, when tested with their caregiver (mother/owner), both dogs and infants that received a positive message moved closer to the object and interacted with it sooner than individuals who had received a negative message [7]–[9], whereas the latter interacted less with the object and showed reduced explorative behaviour [3], [9]–[11]. Hence, using an experimental paradigm closely mirroring that used with children (i.e. conveying the message from the subject's first look and reducing the scariness of the object by switching it off whilst still conveying the message) we found evidence of behavioural regulation in dog–owner dyads. The only substantial difference between our results and those reported in the infant literature is that whereas infants looked more to the mother if she delivered a negative message [7], our dogs looked more often to the owner if s/he delivered a positive message. This pattern was also seen in 6–9 months old infants, who showed referential looking to the mother and an increased duration of looks with a positive rather than a negative message; however, at this age there was no evidence of behavioural regulation, probably due to the infants' inability to detect the fearful affect of the parental communication [7]. In our situation this explanation is unlikely since the dogs behaviour *was* affected by message valence. One potentially important difference between our own and most infant studies, is that whereas children were tested with novel, movable toys, we used an object that was potentially more intimidating for dogs. Hence it is possible that dogs correctly interpreted their owner's encouraging message as an indication to explore the object further but, being uncertain about the object, they looked back more frequently to check that the owner was sure that approaching was a good idea.

Results assessing the effectiveness of the message when delivered by a stranger showed that, although dogs in both message groups looked referentially to the stranger as often as to the owner, they did not approach and interact more with the fan in the positive compared to the negative group. Interestingly, dogs in the negative message group spent more time in the area close to the door (i.e. close to the seated owner), exhibiting more static behaviour and looking more often to the seated owner. Similarly to what has been found with infants, maintaining proximity with the owner may be an expression of comfort-seeking. Taken together these results suggest that probably dogs were sensitive to the emotional expression of the stranger (in line with [24], [47]), but the way they changed their behaviour was dependant on their relationship with the informant. Indeed when a positive message was being conveyed significantly more dogs interacted with the fan if the owner rather than the stranger was the informant. These results are partially in accordance with those emerging from the infant literature. Like our dogs, infants tested with a stranger as the informant, will seek the mother more when receiving a negative message: however, differently from our dogs, they will approach the object more when receiving a positive message from the stranger [12], [15], [16], [48]. There are two possible explanations for dogs' not approaching the object: firstly, as was mentioned above, the stimulus used in infant studies was inherently more attractive, whereas we chose an object which most dogs found a bit intimidating. The motivation to explore it may hence have been quite low, and only be activated by the owner's encouragement. Another possibility is the difference in the owner/mother engagement in the scene. In infant studies, mothers are present and attentive to the interaction that is occurring between the stranger, child and object, whereas in our own study the owner was reading a magazine and facing away from the scene. It is possible that whereas the attentive mother provided infants with enough reassurance that ‘all was well’ when the stranger gave a positive message, the inattentive owner was an element of uncertainty which inhibited dog's potential reaction to the stranger's positive message. Future studies will be needed to address these points, however results from the current study show that although the behaviour of dogs was different depending on informant identity, a clear difference emerged depending on the message sent, showing that dogs were indeed able to distinguish the informant's emotional message.

A possible factor influencing the differential behaviour of dogs in the different groups is the duration of the vocal and facial messages expressed by the informants, however these resulted to be similar across all four groups.

Another possibility is that dogs were affected by the general mood of the informant (and more specifically the owner), rather than understanding that the emotional message referred to a specific object. Mood modification (*sensu* [17]) is a process by which the observer is affected by the emotions of the actor and hence mirrors those same emotions [49]. Whereas a number of infant studies devised experimental paradigms to tease these processes apart [17], the current study did not set out to do so. However, it should be noted that, when tested with the owner, the behavioural changes enacted by dogs could potentially have been directed either at the object or the seated stranger. If dogs had not been sensitive to the referential nature of their owner's communication we would have expected an increased interaction with the seated person in the positive group, and avoidance in the negative group but this was not the case: dogs' behavioural changes were specifically directed to the fan and the area around it.

Finally, results appear interesting also in relation to debates about ‘joint attention’. According to a number of authors gaze alternation behaviour manifested by the subject between the object and the sharer of attention is a necessary but also sufficient condition to show joint attention [50]. Hence, according to this view, in a social referencing paradigm, infants (and in our case dogs) show joint attention towards the object with the caregiver who comments on it. However, more recently, a number of researchers have redefined joint attention, by emphasizing the ‘jointness’ aspect [51], [52]. According to these authors attending to the same thing that one's partner is attending to is not enough for joint attention; rather there needs to be (i), a motivation to share attention and interest with others with no other more instrumental goal; and (b), that both individuals know together that they are sharing attention. According to this view social referencing does not necessarily require joint attention, since the subject may simply exploit the knowledge of the informant without necessarily being engaged in sharing attention with him/her, i.e. without the ‘knowing together’ element of joint attention. In the current study we adopted a stringent definition of gaze alternation, requiring dogs to carry out a 3-way behaviour (fan-informant-fan, or informant-fan-informant) and, although we did not set out to test the ‘jointness’ hypothesis, there may be a number of elements of interest relating to it. Firstly, the motivation behind the dogs' gaze alternation behaviour in general could not be considered a desire to obtain the object since dogs were somewhat intimated by it. Secondly and more importantly, there was an active search on the dogs' part to involve the owner when s/he was inactive by gaze alternating between him/her and the fan. If dogs simply wanted owners to attend to them, they did not need to gaze alternate towards the object, other attention-getting behaviours or gazing to the owner alone would have been sufficient. Taken together these results seem to suggest that dogs “wanted their owner*”* to attend to the same object they were attending to, possibly because the stranger's feedback was not sufficient or relevant enough for them. Third, a different pattern of gaze alternation was evident with the owner and the stranger depending on his/her attentional stance. Dogs gaze alternated more frequently when the person was the informant and hence was also gaze alternating between them and the object than when s/he was seated and inattentive, suggesting that they could recognize when this behaviour was mutual. In light of these preliminary results it will be very interesting to design studies capable of teasing apart the motivation behind dogs' human-directed looking behaviour.

In sum, the current study shows that dogs look back not just to request for a desired object/food but also to check their owner's (but also a stranger's) reaction to an ambiguous object. Furthermore, it is the first study to show that dogs will modify their behaviour towards an object depending on the informants' positive vs. negative message. Hence, dogs use social referencing in their interactions with humans, but when confronted with a potentially scary object, their behaviour towards it seems to be selective and dependent on the relationship with the informant.

## Supporting Information

Text S1 Breed of participating dogs.
(DOC)
