# Supplementary material for: Dogs' Social Referencing towards Owners and Strangers
Source: PLoS One. 2012 Oct 11;7(10):e47653. doi: 10.1371/journal.pone.0047653 (PMC3469536; doi:10.1371/journal.pone.0047653)
Supplement: Text S1 — Breed of participating dogs. (DOC) [file pone.0047653.s001.doc]

**Breeds of participating dogs**

Poodle 1, Dachshund 1, Beagle 2, Border Collie 8, Boxer 1, French Bulldog 2, Czechoslovakian wolfdog 1, Cavalier king Charles 1, Cocker Spaniel 1, Cane Corso 1, Dalmatian 1, Epagneul Breton 1, Flat Coated Retriever 1, Golden Retriever 2, Hovawart 2, Siberian Husky 1, Jack Russell Terrier 7, Labrador Retriever 6, Lagotto Romagnolo 1, Belgian Malinois 1, Maltese 2, German Shepherd 3, Dobermann Pinscher 4, Rhodesian Ridgeback 2, Rottweiler 2, Irish Setter 1, Shih - Tzu 1, Schnauzer 1, American Staffordshire Terrier 1, Pomeranian 1, West Highland Terrier 1.
